# The effect of different angle kappa on higher-order aberrations after small incision lenticule extraction

**DOI:** 10.1007/s10103-023-03934-8

**Published:** 2023-11-28

**Authors:** Lu Guo, Zixuan Cheng, Xiangmei Kong, Zhaoxia Huang, Xue Xu, Jinchuan Wu, Hongbin Lv

**Affiliations:** 1 The First Affiliated Hospital of Jinan University, Guangzhou, 510000 Guangdong Province China; 2 The Affiliated Hospital of Southwest Medical University, Luzhou, 646000 Sichuan Province China

**Keywords:** Angle kappa, Higher-order aberrations, Small incision lenticule extraction (SMILE)

## Abstract

This study aimed to compare higher-order aberrations (HOAs) after small incision lenticule extraction (SMILE) in patients with different angle kappa. This is a retrospective report in which 341 right eyes of 341 patients who were subjected to SMILE, which used coaxially sighted corneal light reflex (CSCLR) as the treatment zone centered, treated by the same experienced surgeon (LHB) for correction of myopia and myopic astigmatism, preoperative and postoperative spherical equivalent (SE), angle kappa, total higher-order aberrations (total HOA), spherical aberration (SA), vertical coma (VC), horizontal coma (HC), oblique trefoil (OT), and horizontal trefoil (HT), were compared. SMILE showed outstanding performance in terms of safety, efficacy, and predictability. In addition, a comparison of preoperative and postoperative HOAs exhibited the difference of total HOA (*P* < 0.01), SA (*P* < 0.01), VC (*P* < 0.01), and HC (*P* < 0.01), which was statistically significant; however, for OT and HT with the longer follow-up time, the statistical difference gradually decreased. For stratification of angle kappa into groups based on decantation, angle kappa was divided into three major groups: *r* < 0.1 mm, 0.1 ≤ *r* < 0.2 mm, and *r* ≥ 0.2 mm; the changes of SA (*F* = 4.127, *P* = 0.021) and OT (*F* = 3.687, *P* = 0.031) exhibited significant difference after 1 year of SMILE. We performed a correlation analysis of all preoperative and postoperative parameters, and the results indicated that the preoperative total HOA was negatively correlated with preoperative cylindrical diopter (DC), and postoperative total HOA, SA, and coma were affected by spherical diopter (DS) and SE. Moreover, we also found a significant difference of SA and VC in the early postoperative with preoperative. SA was positively correlated with *Y* values and *r* of 1 year after SMILE. All of the analyzed parameters in the three groups, except for the trefoil, gradually increased over time; however, the trefoil could gradually stabilize over time. We also divided angle kappa into four groups by quadrants; the result showed that the effects of higher-order aberrations were markedly different from the various quadrants. Patients with large angle kappa were able to increase VC and SA postoperatively, and higher HOAs were more significant in patients with high myopia. The differences in quadrants exhibited a diversity of HOAs; this could be attributed to the corneal surface reestablishment and the alteration of angle kappa, but the trend was not apparent. Although all patients displayed increased HOAs after SMILE, the potential application of CSCLR as the treatment zone centered still showed excellent safety, efficacy, and predictability.

## Introduction

Angle kappa is the primary difference between the pupillary and visual axis; the visual axis refers to a line connecting the point of fixation, the nodal point of the eye, and the center of the fovea [1]. The pupillary axis of the human eye refers to a line which is perpendicular to the center of curvature of the anterior corneal surface and can connect to the center of the pupil. The angle kappa is an important parameter required for the precise treatment of corneal refractive surgery as well as the calculation of the correction amount of strabismus surgery [2–4]. Femtosecond laser-assisted small incision lenticule extraction (SMILE) has demonstrated adequate safety, efficacy, predictability, and stability when used for the correction of myopia and myopic astigmatism [5–8]. The design of the optical area of the visual axis has been demonstrated to be more important for the postoperative visual quality than the pupillary axis during the surgical treatment of myopic patients. At present, the SMILE treatment device in our hospital is not equipped with the scanning tracking system; hence, it is particularly important to conduct accurate preoperative and intraoperative positioning and maintain the size of preoperative angle kappa of patients. A number of previous studies have reported that the angle kappa can mainly affect the postoperative higher-order aberrations (HOAs) in Lasik patients [9, 10], but the potential effect of different-sized angle kappa on postoperative HOAs in patients remains unclear. Therefore, this study mainly analyzed the total higher-order aberrations (total HOA), spherical aberration (SA), vertical coma (VC), horizontal coma (HC), oblique trefoil (OT), and horizontal trefoil (HT) before and after operation with the different angle kappa size (*r*) to obtain an optimal preoperative design of SMILE.

## Patients and methods

### Patients

In this retrospective case study, 341 patients (341 right eyes) who underwent SMILE surgery at the Affiliated Hospital of Southwest Medical University from 2019 to 2021 were selected. All patients were subjected to routine preoperative examinations and met the surgical indications for SMILE. The inclusion criteria used were as follows: (1) 18–35 years old; (2) spherical equivalent (SE) of −1~−10 diopters (D); (3) best corrected visual acuity (BCVA) ≥0.8, stable refraction for 2 years prior to surgery, and the absence of other pathological ocular conditions or relevant systemic diseases; and (4) postoperative residual stromal thickness (RST) ≥280 μm. All patients were advised to stop wearing contact lenses for at least 4 weeks prior to the surgery.

### Methods

All surgeries were performed by the same experienced surgeon (LHB) using the VisuMax femtosecond laser system (Carl Zeiss Meditec AG, Jena, Germany). The parameters used for femtosecond scanning were a repetition rate of 500 kHz, pulse energy of 130 nJ, and intended cap thickness of 110 to 130 μm. The target refraction in all eyes was plano. The width of the side cut was set at 2 mm at the superior 11 o’clock position, and the intended diameter of the lens (optical zone) was set to 6.5 to 7.0 mm. The coaxially sighted corneal light reflex (CSCLR) was set as the center of the treatment zone. We used a Pentacam scan to measure the displacement of the visual axis from the pupil center (chord distance), which essentially equals the angle kappa (*r*), and the angle kappa values were stratified into groups according to the decantation (*r* < 0.1 mm, 0.1 ≤ *r* < 0.2 mm, and *r* ≥ 0.2 mm), and HOAs (total HOA, SA, VC, HC, OT, and HT) were collected from the Pentacam database. Based on the results of the Pentacam scan, the relative positions of the corneal vertex and the pupil center (angle kappa) were obtained before the SMILE surgery. The center of the suction ring was adjusted before the suction, after which the substrate was cut, with blunt separation as well as dissociation of the lens through the incision, and the lens was removed. Finally, the pocket was rinsed with a balanced salt solution to remove the debris. All procedures were completed successfully, and no intraoperative or postoperative complications were observed.

### Follow-up

The postoperative follow-up was conducted at 1 month, 3 months, 6 months, and 12 months. The following data were obtained: SE, angle kappa, total HOA, SA, VC, HC, OT, and HT, which were measured by Pentacam before and after SMILE.

### Statistical analyses

Statistical analyses and graphics were performed using SPSS 26.0 and GraphPad Prism 8.0.1, respectively. The distributions of the numerical variables (total HOA, SA, VC, HC, OT, and HT) were expressed by the Skewness and Kurtosis coefficient. If the absolute value of skewness was less than 3 and the absolute kurtosis value was less than 10, the distribution was considered normal or approximately normal [11]. Continuous data are presented as mean ± standard deviation, and categorical data as frequencies and percentages. Differences between groups of normally distributed data were compared using independent sample *t*-tests, while multi-group comparisons were analyzed by one-way ANOVA. The numerical variables for the postoperative follow-up visits (1 month, 3 months, 6 months, and 12 months after surgery) were compared with the baseline using paired sample *t*-tests. The trends of numerical variables between the groups were compared using repeated ANOVA to explore the possible interaction effects (trends in different categories) and the main effects (points and categories). Pearson correlation coefficients were used to assess associations between normal variables. The significance level was set at 0.05.

## Results

### Preoperative patient demographics and refractive diopters

A total of 341 right eyes from 341 individuals were included in the study, complying with the inclusion criteria of DS (−4.63 ± 1.76), DC (−0.66 ± 0.62), SE (−4.96 ± 1.83), and age (23.26 ± 5.69). All surgeries were successful, and no specific intraoperative complications were observed**.**

### Basic information (Table 1)

We observed that SMILE showed outstanding performance in terms of safety, efficacy, and predictability (both eyes). The uncorrected distance visual acuity (UDVA) increased steadily by more than 1 row after SMILE. All patients had stable vision greater than 1.0 within one year of SMILE. Postoperative DC below 0.5 D accounted for more than 80%.

**Table 1:** Comparison of safety, efficacy, and predictability after SMILE (both eyes) (%)

	1 month post-op	3 months post-op	6 months post-op	1 year post-op
Eyes	444	496	290	123
Safety				
UDVA add more than one row	50	51.2	62	55.3
UDVA drop more than one row	7	9	4	1
Efficacy				
UDVA ≥1.0	98.2	98	98	100
UDVA ≥0.8	99	99.7	98.2	100
Predictability				
Post-op diopter ≤0.5D	81.3	81.6	83.4	85.4
Post-op diopter ≤1.0D	96	96	97.2	99

*UDVA*, uncorrected distance visual acuity

### Comparison of HOAs preoperative and postoperative (Table 2)

The comparison of preoperative and postoperative HOAs showed differences in the total HOA (*P* < 0.01), SA (*P* < 0.01), and VC (*P* < 0.01), as well as HC (*P* < 0.01), all of which were statistically significant. However, the degree of significance of the OT and HT values gradually declined as the follow-up time increased.

**Table 2:** Comparison of RMS of higher-order aberration pre- and post-operation (μm)

Zernike	Pre-op	1 month post-op	3 months post-op	6 months post-op	1 year post-op	Pre vs. 1 month post-op		Pre vs. 3 months post-op		Pre vs. 6 months post-op		Pre vs. 1 year post-op	
						*t*	*p*	*t*	*p*	*t*	*p*	*t*	*p*
Total HOA	1.625±0.481	1.709±0.496	1.854±0.534	1.9278±0.647	1.896±0.489	18.672	0.000**	5.624	0.000**	6.151	0.000**	3.912	0.000**
VC	−0.030±0.172	−0.250±0.258	−0.261±0.259	−0.271±0.266	−0.274±0.253	20.249	0.000**	−16.073	0.000**	−12.258	0.000**	−9.428	0.000**
HC	−0.084±0.093	−0.158±0.170	−0.182±0.199	−0.206±0.189	−0.218±0.144	18.005	0.000**	−8.360	0.000**	−7.811	0.000**	−8.603	0.000**
SA	0.238±0.074	0.357±0.128	0.366±0.122	0.376±0.119	0.361±0.096	19.252	0.000**	17.554	0.000**	14.718	0.000**	9.136	0.000**
HT	−0.010±0.095	0.011±0.116	0.0039±0.121	0.006±0.108	0.007±0.118	17.684	0.000**	2.481	0.014*	0.762	0.447	1.744	0.086
OT	−0.026±0.079	−0.035±0.091	−0.015±0.101	−0.016±0.095	−0.016±0.087	20.538	0.000**	2.195	0.02*	1.327	0.186	0.358	0.721

**p < 0.01

*p < 0.05

### Comparison of HOAs with different preoperative and postoperative angle kappa (Table 3)

Stratification of the angle kappa into groups based on decantation resulted in the division into three main groups, namely, *r* < 0.1 mm, 0.1 ≤ *r* < 0.2 mm, and *r* ≥0.2 mm. The basic data and HOAs for both the preoperative and postoperative time points were compared and analyzed. While no significant differences were observed in the preoperative parameters, significant changes in the SA (*F* = 4.127, *P* = 0.021) and OT (*F* = 3.687, *P* = 0.031) were found one year after SMILE.

**Table 3:** Comparison of higher-order aberrations with different angle kappa preoperative and postoperative

	*N*	*r* < 0.1 mm	0.1 ≤ *r* < 0.2 mm	*r* ≥ 0.1 mm	*P*
Age	227	22.900±7.623	23.032±6.366	24.300±6.798	0.269
DS	227	−4.407±1.826	−4.579±1.604	−4.903±1.918	0.147
DC	227	−0.593±0.460	−0.692±0.754	−0.673±0.464	0.487
SE	227	−4.704±1.886	−4.925±1.709	−5.239±1.957	0.138
Total HOA	227	0.206±0.541	0.014±0.601	0.065±0.619	0.167
VC	227	−0.223±0.257	−0.224±0.191	−0.224±0.213	1
HC	227	−0.065±0.131	−0.078±0.142	−0.091±0.185	0.673
SA	227	0.116±0.105	0.110±0.129	0.124±0.127	0.764
HT	227	0.028±0.101	0.034±0.101	−0.001±0.092	0.074
OT	227	−0.005±0.097	0±0.09	−0.017±0.082	0.452
SA pre vs. 1 year post	227	0.006±0.101	0.111±0.085	0.149±0.095	0.021*
OT pre vs. 1 year post	227	−0.033±0.067	0.035±0.093	−0.010±0.085	0.031*

*p<0.05

### Comparison of changes in HOAs in the different quadrants under both preoperative and postoperative settings (Table 4)

The angle kappa values were divided into four groups according to quadrants, and it was found that the HOAs differed between the various quadrants. No statistically significant differences were found between the quadrants, and although differences were seen in several parameters, these were non-significant.

**Table 4:** Comparison of changes of HOAs in the different quadrants under both preoperative and postoperative settings

	Quadrants	*N*	Mean	Std. deviation	*F*	*P*
HT pre vs. 3 months post	1	103	0.012	0.120	2.775	0.042*
	2	85	0.028	0.097		
	3	35	0.044	0.099		
	4	31	−0.027	0.104		
OT pre vs. 6 months post	1	51	0.042	0.102	3.535	0.016*
	2	58	−0.015	0.096		
	3	24	0.020	0.089		
	4	16	−0.010	0.086		
SA pre vs. 3 months post	1	29	0.118	0.095	3.642	0.017*
	2	25	0.130	0.080		
	3	5	−0.015	0.130		
	4	5	0.097	0.076		

*p < 0.05

### Distribution of the angle kappa in different sizes (Fig. 1)

Stratification of the angle kappa into three groups and a scatter diagram showed that the angle kappa was mainly concentrated in the first and second quadrants, while the *r* < 0.1 (34%) and *r* ≥ 0.2 (49%) groups were found mostly in the second quadrant and the 0.1 ≤ *r* <0.2 (39%) was mainly localized in the first quadrant. Differences in the composition ratios of the quadrants showed that the second quadrant had the highest proportion and the fourth quadrant had the lowest proportion (*χ*^2^ = 21.795, *P* < 0.001).

**Fig. 1:**
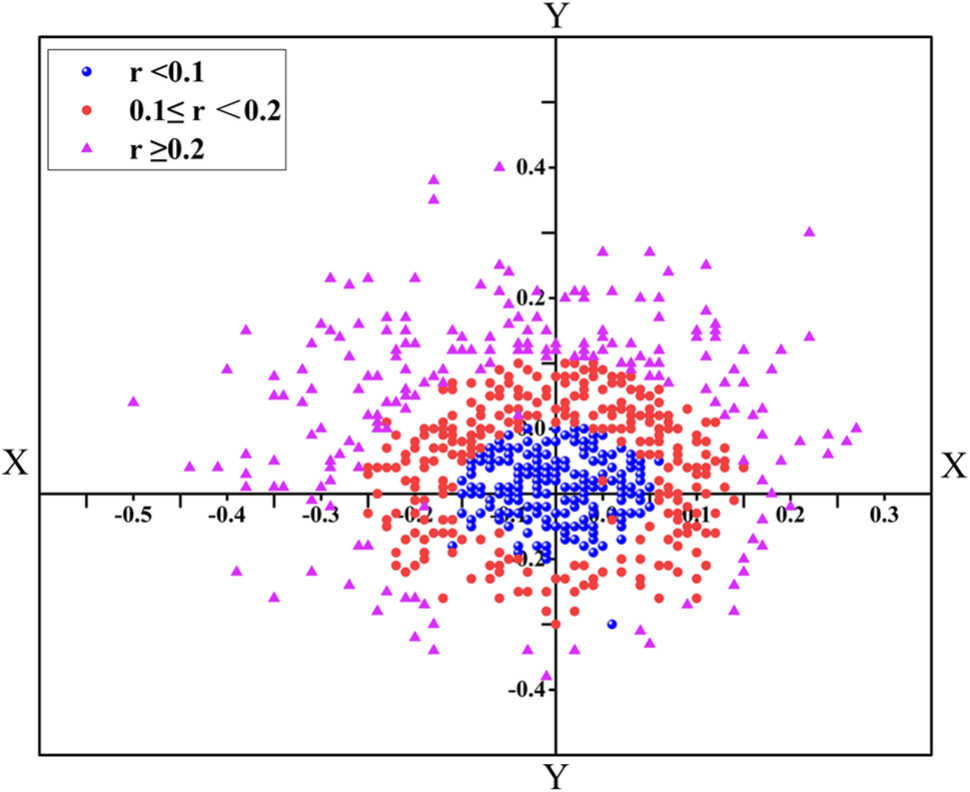
Distribution of angle kappa in different sizes. The scatter diagram showed that the angle kappa was mainly concentrated in the first and second quadrants, the group *r* < 0.1 (34%) and the group *r* ≥ 0.2 (49%) were mainly concentrated in the second quadrant, the group 0.1 ≤ *r* < 0.2 (39%) was mainly localized in the first quadrant

### Correlation analyses (Table 1)

We performed correlation analyses for all preoperative and postoperative parameters. It was observed that preoperative total HOA values were negatively correlated with preoperative DC, and the smaller the DC, the greater the total HOA. In addition, the preoperative values of SA and coma were negatively correlated with both DS and SE at 1 month and 3 months after surgery; the smaller the DS and SE values (the larger were the negative DS and SE values), the greater those of SA and coma. The preoperative total HOA and SA were negatively correlated with DS and SE 6 months after the surgery, with lower DS and SE (larger negative DS and SE values) associated with greater total HOA and SA. The *r* and *Y* values were positively correlated with SA.

**Table 5:** Correlation analysis of  preoperative and postoperative parameters

Variable	DS (pre)	DC (pre)	SE (pre)	*r* (pre)	*Y*-axial (pre)
angle (pre)	0.273	0.116	0.281	−0.182	−0.617
*Y*-axial (pre)	−0.224	−0.096	−0.231	0.083	0.125
Total HOA (pre)	0.058	−0.389	−0.010	0.077	0.034
VC (1 month post)	0.336	0.062	0.334	−0.072	−0.198
SA (1 month post)	−0.466	0.253	−0.420	0.007	0.040
VC (pre vs. 1 month post)	0.305	0.120	0.312	−0.032	−0.134
SA (pre vs. 1 month post)	−0.534	0.174	−0.496	0.034	0.088
VC (pre vs. 1 month post)	−0.381	−0.171	−0.392	0.008	0.141
HC (pre vs. 1 month post)	−0.314	−0.064	−0.313	0.048	0.102
SA (pre vs. 1 month post)	−0.486	0.119	−0.456	0.058	0.141
SA (3 months post)	−0.471	0.202	−0.434	−0.029	0.054
Total HOA (pre vs. 3 months post)	−0.324	0.382	−0.265	−0.017	0.020
SA (pre vs. 3 months post)	−0.554	0.151	−0.522	−0.001	0.076
Total HOA (6 months post)	−0.330	0.127	−0.286	−0.032	0.088
SA (6 months post)	−0.535	0.002	−0.505	−0.052	0.061
Total HOA (pre vs. 6 months post)	−0.330	0.289	−0.254	−0.091	0.043
HC (pre vs. 6 months post)	0.397	0.091	0.394	−0.035	−0.053
SA (pre vs. 6 months post)	−0.601	−0.082	−0.584	−0.037	0.049
SA (pre vs. 1 year post)	−0.360	−0.081	−0.360	0.274	0.315
Total HOA (pre vs. 1 year post)	−0.207	0.516	−0.130	−0.063	−0.082
SA (pre vs. 1 year post)	−0.508	−0.064	−0.501	0.357	0.354

### Trends of individual aberrations between the different angle kappa sizes (Fig. 2)

No differences were observed between the groups (*P > 0.05*), and all parameters in the three groups, except for the trefoil, increased gradually over time, while the trefoil remained stable over time.

**Fig. 2:**
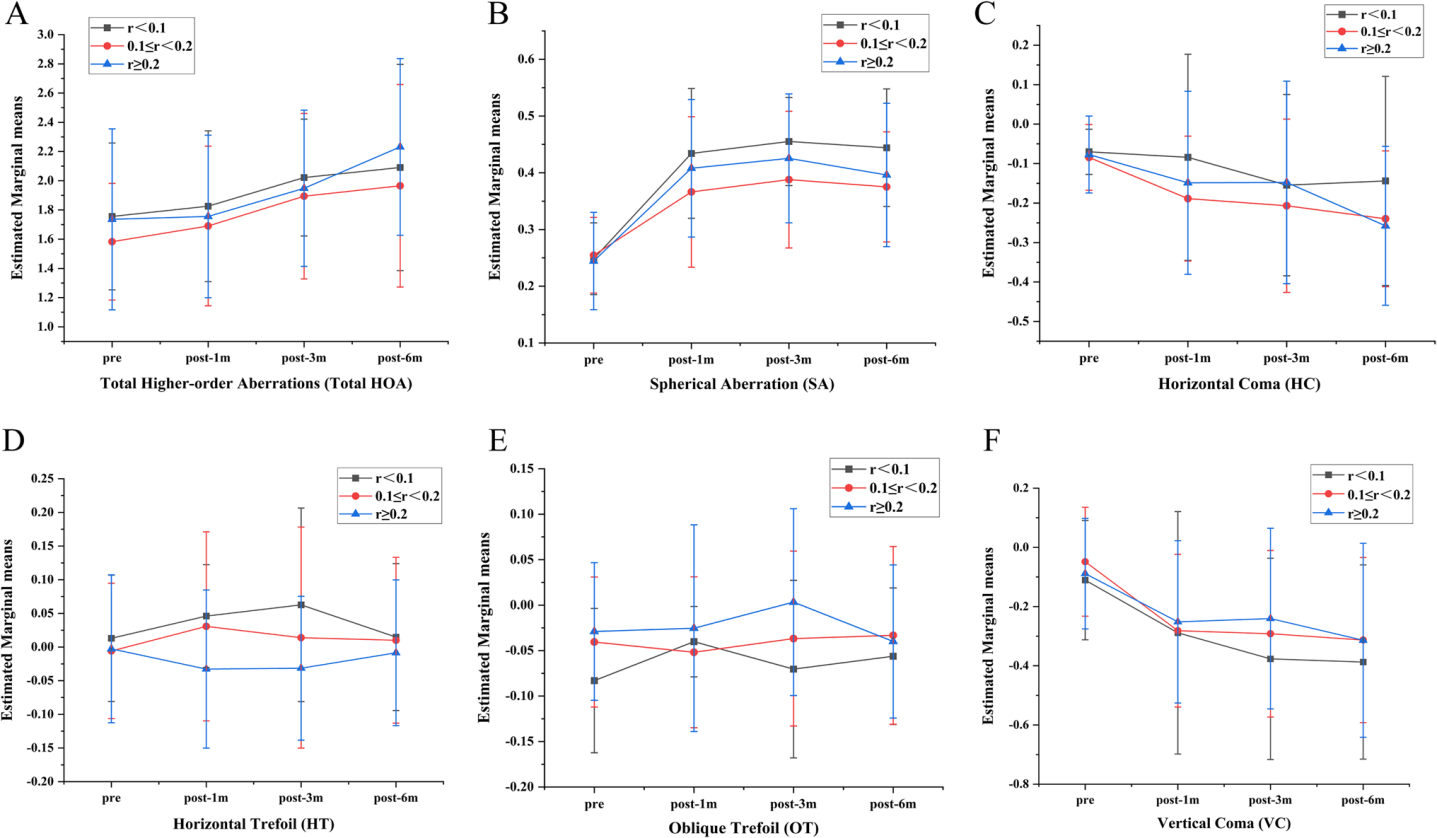
Trends of individual aberrations between the different angles of kappa size. There were no differences between groups (*P > 0.05*); all parameters of the three groups, except for the trefoil, increased gradually over time (ABCF); however, the trefoil (DE) was kept stable over time

## Discussion

The techniques of refractive surgery are evolving with rapid, ongoing advances in research. It has been established that a treatment zone centered at the visual axis, instead of the pupillary axis, is the key to optimizing the visual outcome of a refractive surgery [12, 13]. At present, in corneal refractive surgery, the cutting of the corneal center is divided into four distinct methods, namely, the center of the pupil (PC) [14], CSCLR [15], corneal vertex normal (CVN) [16, 17], and between the pupillary and visual axis [18]. However, there is controversy regarding optimal centration in corneal refractive procedures. In other words, in the virtual image of the light source, also known as the first Purkinje-Sanson image, the corneal light reflex is formed by the reflection of light from the anterior corneal surface. A number of researchers have postulated that the CSCLR from the cornea lies closer to the corneal intercept of the visual axis than the PC and have thus recommended using the corneal CSCLR as the center of the refractive surgery [19]. However, the CSCLR was used as the surgical center for all patients in the present retrospective study, with adjustment of the angle kappa, and all patients were satisfied with the results. In addition, SMILE showed good safety, efficacy, and predictability.

HOAs are optical imperfections of the eye that alter the quality of the retinal image despite optimal correction of spherical defocus and astigmatism. Since HOAs reduce the retinal image quality and produce variations in optical vergence across the entrance pupil of the eye, they may provide optical signals that contribute to the regulation and modulation of eye growth and refractive error development. The third-order coma and trefoil and fourth-order spherical aberration constitute the major components of HOAs, with the coma and trefoil aberrations considered variations of magnification with respect to the aperture. If the paraxial magnification is equal to the real ray marginal magnification, then an optical system would be free of coma or trefoil. Spherical aberration can be considered a variation of the focal length with the aperture. These aberrations can cause difficulty in seeing at night, glare, halos, blurring, starburst patterns, or double vision (diplopia). In this report, all HOA parameters were compared before and after SMILE by Pentacam, and all exhibited significant differences except trefoil (although with longer follow-up times, the statistical difference gradually decreased). This result indicated that trefoil could be stabilized over time.

Several prior studies [20, 21] have indicated that the angle kappa decreases with age, although the changes in its direction or value are not significant. Hashemi [22] reported that the angle kappa decreased by about 0.015 per year increase in age. In this report, we divided the angle kappa into three distinct groups, namely, *r* < 0.1 mm, 0.1 ≤ *r* < 0.2 mm, and *r* ≥ 0.2 mm, finding that there was no significant difference between the groups with age. The reason can be attributed to the fact that the inclusion criteria included age (mean 23.377 ± 6.816); therefore, a valid comparison of age could not be made. Secondly, there were significant differences in SA and OT before and 12 months after SMILE, which implies that the early differences in HOAs after SMILE surgery were not significant in patients with different angle kappa, while some may change after 12 months, especially SA in the group with the larger angle kappa. This may have been caused primarily by the aspherical reconstruction of the corneal surface after partial corneal central cutting. Thirdly, there were no significant differences between the groups in terms of DC, DS, and SE, a finding that was surprisingly quite different from those of previous reports. For instance, Basmak [23] found that the angle kappa decreased significantly with increasing DS. Ding [24] reported that a large angle kappa was observed mostly in patients with medium and low myopia, while a small angle kappa was mostly associated with high myopia, as patients with high myopia often need to move close to objects to see them clearly. Clinically, these changes show a significant shortening of the near point distance, and the eye three-linkage effect of the near-reflection system could be stimulated (accommodation, convergence, and pupil shrink) to maintain monocular function in both eyes. In addition, the binocular convergence function must be strengthened, i.e., the inward rotation of the eyeball, so that high myopia can exhibit a small angle kappa. The reasons for the different conclusions in this study can be possibly attributed to insufficient sample size, together with the predominance of patients with middle and low myopia, with patients with high myopia accounting for only 21%, which may have caused statistical bias and led to this peculiar trend.

To better understand whether the orientation of the angle kappa affected the postoperative HOAs, the angle kappa values were divided into four distinct groups according to quadrant (Table 4). The result showed that there were no statistically significant differences between the quadrants. However, a few changes were observed which may have been due to wound healing and the postoperative reestablishment of the aspheric vertices on the corneal surface, resulting in alteration of the angle kappa [24]. At the same time, we drew a scatter plot of the angle kappa distribution (Fig. 1), which showed that the angle kappa was mainly concentrated in the first and second quadrants, as the fovea was located on the pupil axis and slightly temporal to the posterior pole, resulting in the normal angle kappa being slightly positive.

We performed correlation analyses on all preoperative and postoperative parameters. The results showed that higher degrees of myopia were associated with greater changes in the total HOA, SA, and coma. We also found that patients with large angle kappa values showed greater increases in SA after the operation; this may have been due to postoperative aspherical changes in the corneal surface, forming a new off-axis center and altering the postoperative angle kappa. The results showed significant differences in SA and VC between the preoperative and early postoperative periods, which can be explained by the technique itself [25]. The patients’ side cuts were designed in the vertical direction, and healing of the surgical incision and the resultant scar formation could have led to an increase in the VC. Thus, changes in asphericity and the gradient refractive index of the corneal surface after SMILE can potentially cause an increase in SA.

We performed a tracking trend analysis of HOAs in the different groups of patients. The results showed the greatest changes in all HOAs during the first month, after which the trend gradually slowed; however, no significant differences were observed between the three groups. Although increases in HOAs were seen in all patients, the patients nevertheless showed excellent visual quality, with good postoperative refractive outcomes obtained in all groups, which may be because the angle kappa is generally small in myopic eyes and had been adjusted. Thus, the postoperative decentration in the eyes of the large angle kappa group was not sufficient to affect the visual and refractive low-order aberration outcomes. HOAs can only affect patients in certain situations, such as having larger pupils at night, potentially leading to the experience of glare, diplopia, and reduced contrast sensitivity. In 2003 [26], a case report of a patient with a large bilateral angle kappa provided the first direct comparison of centering on the entrance pupil versus centering on the CSCLR. The patient had undergone LASIK centered on the entrance pupil in the right eye and over the CSCLR in the left eye. The left eye demonstrated significantly better visual acuity, smaller refractive error, and a smaller amount of postoperative decentration. Kermani [18] did report a significant decrease in the total HOA in the CSCLR group, and this effect was not noted in the pupil-centered group. Moreover, in the same year, Okamoto [27] compared myopic LASIK centered on the CSCLR with centration on the entrance pupil in 556 eyes with unknown angle kappa values. LASIK centered on the CSCLR was found to be significantly safer, more effective, and had lower induction of coma, as well as total HOA, in comparison to LASIK treatments centered on the pupil. However, in 2011, Soler [28] published the only randomized double-masked comparison of the pupil-centered vs. corneal reflex-centered hyperopic LASIK, and the findings of this study concluded that there were no statistical differences. In 2013, Reinstein [19] compared different angle kappa values using the CSCLR treatment, finding no significant differences in safety, accuracy, induced astigmatism, contrast sensitivity, or night vision disturbances after the surgery. These findings are consistent with our results. However, these authors did not conduct further comparative analysis of HOAs, and the results of our report thus provide the clinical basis for our next prospective study. This is primarily because the size of the angle kappa showed no significant effect on the result following the CSCLR treatment, and considering the accuracy of the small kappa angle correction, later prospective studies may consider including large angle kappa for further investigation.

The small angle kappa in SMILE surgery may not need to be adjusted, but there are very few studies describing the correction of the angle kappa after SMILE; nevertheless, large angle kappa must be adjusted to obtain better visual quality. This report indicated that patients with large angle kappa were able to increase VC and SA due to the aspheric and gradient refractive indices of the corneal surface, as well as healing of the early incision and scar formation. Although all patients had increased HOAs after SMILE, the procedure still showed excellent safety, efficacy, and predictability. In this study, the rate of patient follow-up declined between 6 months and 1 year after surgery, which may have had a certain influence on the results. Nevertheless, based on the surgical treatment and observation standard [29, 30], the patients were essentially recovered and stable within 3 months after surgery and drug treatment had ceased. In addition, to avoid missing patients with poor postoperative results, we conducted long-term telephone and network follow-ups, in which none of the patients reported visual-related discomfort or abnormalities. Therefore, the results of this study have some reference value.

This is the first study comparing the effects of the different quadrants of angle kappa on HOAs. Although the different quadrants showed some differences in HOAs, these were non-significant. It is possible that due to the corneal surface reestablishment and change of diopter of myopia after SMILE surgery, the three-linkage effect of the near-reflection system was reduced, and thus, the angle kappa was reconstructed as well as altered; however, this trend was not apparent. The current study was limited by its design and small sample size. We also did not evaluate subjective visual quality and symptoms, such as contrast sensitivity, halo, and glare, in our patients. Thus, further studies with larger sample sizes and diopter differences are still needed, which can also evaluate the potential effect of angle kappa adjustment on light scattering, visual quality, and contrast sensitivity.
